# Intracellular Pharmacokinetics of Antidiabetic Drugs: A Focused Narrative Review of Subcellular Distribution (2015–2025)

**DOI:** 10.3390/diseases14020062

**Published:** 2026-02-09

**Authors:** Duaa Abdullah Bafail

**Affiliations:** Department of Clinical Pharmacology, Faculty of Medicine, King Abdulaziz University, Jeddah 21589, Saudi Arabia; dbafail@kau.edu.sa

**Keywords:** antidiabetic drugs, intracellular pharmacokinetics, subcellular distribution, precision diabetology, drug transporters, mitochondria, regulatory science

## Abstract

**Background/Objectives:** The efficacy of antidiabetic drugs is determined by intracellular target exposure rather than solely by plasma concentrations. This review synthesizes current evidence regarding subcellular drug distribution and its clinical significance. **Methods:** A structured review of the literature published between 2015 and 2025 identified 73 relevant studies. Data were categorized by drug class, factors influencing distribution, and analytical methodologies. **Results:** Drug distribution patterns differ by class. Biguanides accumulate in mitochondria, thiazolidinediones localize in cell nuclei, and GLP-1 agonists are found in endosomes. Variations in transporter genes, such as OCT1, influence the extent of drug delivery to these subcellular targets. **Conclusions:** Investigations into intracellular drug movement elucidate their mechanisms of action. However, standardized human studies are required before these findings can inform clinical practice or regulatory decisions.

## 1. Introduction

Type 2 diabetes mellitus (T2DM) remains a profound global health challenge, affecting over 529 million adults in 2021, with projections reaching 9.8% prevalence by 2050, driven primarily by rising obesity [1,2]. The pharmacological management of T2DM involves a broad range of drug classes that exert their effects through diverse mechanisms across multiple tissues [3,4]. However, a critical gap exists between standard clinical practice and the mechanistic understanding of drug action: therapeutic decisions are predominantly guided by plasma drug concentrations, whereas actual exposure at relevant subcellular targets is rarely measured or considered [5,6].

For example, metformin dosing does not account for OCT1 polymorphisms (rs12208357) that reduce hepatic uptake by 50%, causing a poor glycemic response in 20–30% of T2DM patients despite normal plasma metformin levels [7,8,9,10]. Similarly, thiazolidinediones (TZDs), such as rosiglitazone, require nuclear peroxisome proliferator-activated receptor γ (PPARγ) accumulation for efficacy; however, in the PROactive trial, plasma PK monitoring failed to predict cardiovascular outcomes [11,12]. These clinical cases demonstrate how plasma-focused practices do not address the subcellular drivers of response [5,6].

The discipline of intracellular pharmacokinetics (PK) offers an essential framework for bridging this gap. It connects a drug’s concentration at its specific site of action within the cell to its ultimate pharmacological effect [5,6]. The distribution of a drug within a cell is not passive; it is actively regulated by the drug’s physicochemical properties, its interactions with membrane transporters, and the distinct microenvironments of individual organelles [13,14]. These principles explain why different antidiabetic classes have distinct subcellular destinations, such as the mitochondrial accumulation of biguanides or the nuclear localization of thiazolidinediones, which are fundamental to their mechanisms of action.

Despite the development of sophisticated techniques for visualizing and quantifying intracellular drug distribution, a significant translational gap persists. Regulatory guidelines and clinical trial endpoints continue to prioritize systemic pharmacokinetics, leaving the subcellular drivers of efficacy and toxicity poorly characterized in a clinical context [5,6]. This narrative review aims to synthesize and critically evaluate the existing literature on the intracellular PK of antidiabetic drugs. To provide a comprehensive overview, we will first outline the fundamental principles that determine subcellular distribution. We then survey the key methodological advances that enable the study of these processes. A major focus will be a detailed, class-by-class analysis of the intracellular disposition of major antidiabetic agents. Finally, we will discuss the clinical and regulatory implications of integrating intracellular PK into the precision diabetology paradigm.

## 2. Materials and Methods

Web of Science was searched from database inception to 31 August 2025 [15,16]. Search techniques combined terms related to intracellular pharmacokinetics and subcellular processes (e.g., “intracellular pharmacokinetics,” “subcellular distribution,” and “organelle targeting”) with antidiabetic-specific keywords, including drug classes and individual agent names.

Eligible publications included original research articles, reviews, and clinical studies that reported data on the intracellular or subcellular disposition of antidiabetic drugs (73 studies were included from 1247 records screened, 2015–2025) [17,18]. Studies were prioritized if they used robust and explicitly described methodologies for quantifying subcellular drug distribution, such as validated LC–MS/MS fractionation, high-resolution imaging, or advanced biophysical approaches.

The quantitative values were extracted from the included studies. For each publication, data were categorized by antidiabetic drug class investigated and the primary experimental method used to assess intracellular pharmacokinetics, and frequencies were counted manually from explicit descriptions in the original reports.

## 3. Results and Discussion

### 3.1. Determinants of Intracellular Distribution

#### 3.1.1. Conceptual Framework of Intracellular Pharmacokinetics

Intracellular pharmacokinetics (PK) describes the distribution of drugs over time and space within cells, impacting their ability to reach specific molecular targets and produce pharmacological effects. Unlike systemic PK, which examines drug distribution throughout the entire body, intracellular PK is influenced by interactions between a drug’s physicochemical properties, membrane transport systems, and the distinct microenvironments of individual organelles [5,19].

#### 3.1.2. Physicochemical Determinants

The physicochemical properties of a drug significantly influence its distribution. Important factors, such as molecular weight, lipophilicity (logP), and ionization constant (pKa), determine passive diffusion across lipid bilayers rather than transporter-mediated uptake [20,21]. Small molecules with moderate lipophilicity tend to exhibit better membrane permeability, whereas larger, more polar compounds are more readily transported via facilitated transport [22,23]. Excessive lipophilicity can lead to nonspecific membrane binding, thereby decreasing bioavailability [22,23]. The key physicochemical factors are listed in Table 1.

The ionization state of a drug at physiological pH significantly affects its membrane permeability and interactions with transporters. Weakly basic compounds, in which pKa determines the balance between charged and uncharged forms, cross membranes mainly in their neutral form [24,25]; however, when entering acidic organelles, such as lysosomes (pH ≈ 5.0), protonation leads to trapping—known as lysosomotropism [26,27].

Drug distribution is further modified by organelle abundance, composition, and membrane potential gradients across mitochondria/lysosomes [28,29]. Recent Nernst–Planck models incorporating electrochemical gradients have improved the prediction of ionized species diffusion [30,31].

Lysosomotropism occurs in cationic amphiphilic drugs and is linked to antidiabetics, particularly DPP-4 inhibitors [32,33], where lysosomal accumulation prolongs cellular retention but raises toxicity concerns. Emerging biomimetic carriers (extracellular vesicles) and integrin-targeted conjugates modulate this trafficking for tissue-selective delivery [34].

#### 3.1.3. Transporter-Mediated Distribution

**Figure 1:**
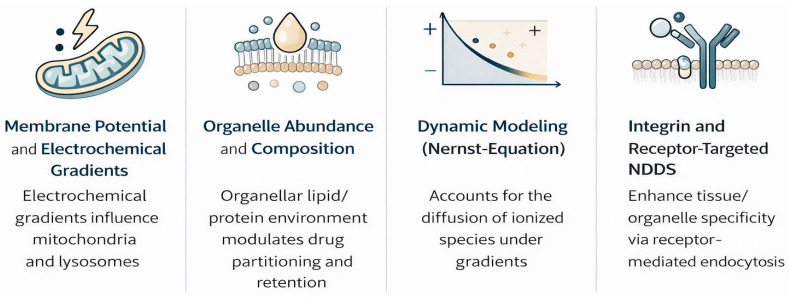
Key physicochemical and biological factors influencing the intracellular and subcellular distribution of antidiabetic drugs, including membrane permeability, transporter activity, organelle membrane potential, vesicular trafficking, and intracellular binding. These processes govern drug access to mitochondria, lysosomes, nuclei, and endosomal compartments.

In the case of many antidiabetic medications, the level of drug accumulation within cells is primarily determined by dedicated transport mechanisms rather than simple diffusion across membranes (Figure 1). These mechanisms include influx transporters that facilitate entry and efflux transporters that promote expulsion, collectively controlling how drugs are absorbed, held within cells, and cleared. Key players in this process include organic cation transporters (OCTs) [35], multidrug and toxin extrusion proteins (MATEs) [36], and the ATP-binding cassette subfamily B member 1 (ABCB1), commonly known as P-glycoprotein [37]. The functionality of these transporters influences both the drug’s access to specific intracellular sites and the reasons for variations in patient responses. A clear illustration of this reliance is metformin, a water-soluble cationic biguanide compound. It depends on OCT1 (encoded by SLC22A1) to enter liver cells, allowing its buildup within them and directing action against Complex I of the mitochondrial electron transport chain [38]. Differences in metformin treatment outcomes have been associated with genetic variants in OCT1 that impair its function, leading to decreased liver uptake and diminished blood sugar control. This evidence underscores the role of transporter genetics in shaping drug performance.

In contrast, efflux mechanisms add further regulation. For instance, P-glycoprotein (ABCB1) expels drugs from intestinal and hepatic cells, thereby limiting their intracellular accumulation. Elevated levels of these efflux proteins can diminish drug potency, whereas blocking them might prolong cellular exposure at the expense of increased adverse effects. This interplay between influx and outflow mechanisms highlights the importance of evaluating transporter–drug pairings in antidiabetic treatments. Another example involves peptide therapeutics that introduce a distinct pathway [39]. Glucagon-like peptide-1 (GLP-1) receptor agonists, which are significantly bulky polar molecules that cannot permeate membranes, are taken up by endocytosis triggered by receptor binding. The resulting receptor–ligand complex enters endosomes, where it sustains signaling activity. This process supports the prolonged effects of these agonists and shows that transporter dynamics extend to larger biomolecules beyond traditional small-molecule drugs [40]

#### 3.1.4. Organelle-Specific Accumulation

After cellular uptake, drugs often localize selectively to organelles, influencing efficacy or toxicity in antidiabetic treatments. Mitochondria are a key site where the inner membrane’s negative potential attracts lipophilic cations. Metformin exemplifies this process: it enters hepatocytes via OCT1, accumulates in mitochondria to block Complex I, lowers ATP, elevates AMP/ATP ratios, and activates protein kinase (AMPK) [41], thus suppressing gluconeogenesis and enhancing insulin sensitivity. However, overaccumulation contributes to the risk of lactic acidosis. Lysosomes trap weakly basic drugs in their acidic interior via ion trapping, prolonging retention. Some DPP-4 inhibitors exhibit this, creating a reservoir for sustained action but potentially limiting availability at other sites and impairing lysosomal function over time [42]. Thiazolidinediones (TZDs), being lipophilic, penetrate the nucleus, bind PPARγ, and modulate genes for glucose and lipid handling critical for insulin sensitization, although they are linked to cardiovascular risks from excess nuclear activity [43] (Figure 1).

Remarkably, some antidiabetics bypass organelles: SGLT2 inhibitors target the renal membrane [44], and many DPP-4 inhibitors act on extracellular or membrane enzymes. This variability shows that subcellular distribution is vital for some inhibitors but unnecessary for others.

#### 3.1.5. Additional Determinants of Subcellular Distribution

In addition to physicochemical and transporter mechanisms, other factors also modulate intracellular distribution. Intracellular protein binding influences free drug availability; metformin exhibits low plasma binding (<10%) but high mitochondrial Complex I affinity [38]. Organelle metabolic activity affects retention—lysosomal enzymes metabolize DPP-4 inhibitors like linagliptin, creating sustained-release reservoirs [45].

DNA/organelle binding drives nuclear accumulation; TZDs bind PPARγ-DNA complexes, prolonging transcriptional effects [43]. Organelle membrane permeability varies; that is, the mitochondrial inner membrane restricts hydrophilic drugs, while ER membranes favor lipophilic agents [41]. Vesicular transport governs peptide drugs; GLP-1 receptor agonists undergo receptor-mediated endocytosis into endosomes, permitting prolonged signaling [40].

## 4. Methodological Approaches for Quantifying Intracellular PK

Assessing the pharmacokinetics (PK) of a drug within cells requires targeted techniques to determine both the location and quantity of drug accumulation. Existing strategies are grouped into three main types, namely imaging methods, organelle separation paired with quantitative analysis, and sophisticated biophysical techniques, each offering unique benefits and certain drawbacks [46,47] (Table 2).

### 4.1. Imaging-Based Methods (HCI, Confocal Microscopy, and Fluorescent Probes)

**Figure 2:**
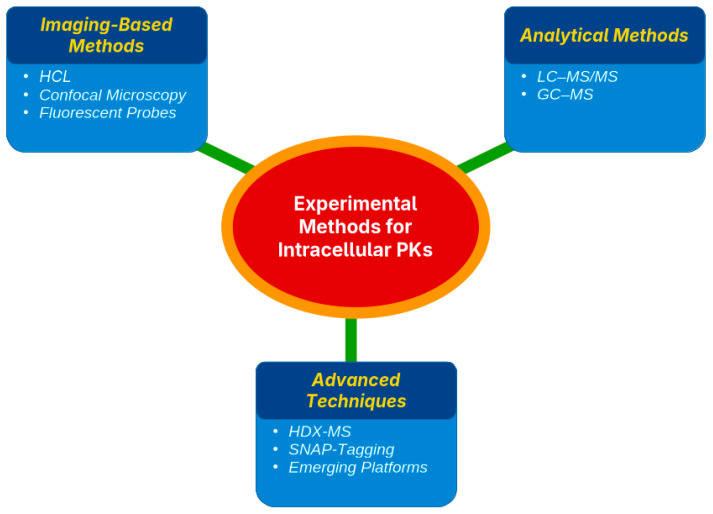
Principal experimental strategies used to assess intracellular pharmacokinetics, including imaging-based methods, LC–MS/MS following subcellular fractionation, and advanced biophysical and mass spectrometry-based techniques, with differing spatial resolution and quantitative capacity.

Imaging techniques visualize drug distribution in live cells using fluorescently tagged drugs and probes. Confocal laser scanning microscopy (CLSM) was used to track subcellular localization [48,49]. For example, GLP-1 receptor agonists traffic to endosomes or DPP-4 inhibitors accumulate in lysosomes [50,51]. High-content imaging (HCI) automates image capture and analysis for high-throughput studies, tracking insulin analogs, GLP-1 RAs, or drug-induced mitochondrial stress in β-cells [52,53]. These methods offer high spatial resolution and dynamic insights into drug–target co-localization using organelle-specific probes. However, fluorescent tagging is often required, which may not always be possible, and CLSM has low throughput [54,55] (Figure 2).

Imaging typically provides qualitative or semi-quantitative data (fluorescence intensity) rather than absolute concentration data. Despite this, imaging is vital for mapping drug uptake and retention dynamics, such as rapid GLP-1 receptor endocytosis observed via CLSM.

### 4.2. Fractionation and Analytical Methods (LC–MS/MS, GC–MS)

Subcellular fractionation isolates organelles (e.g., mitochondria, lysosomes, and nuclei) by lysing cells, performing differential centrifugation, and quantifying drugs in each fraction [56,57]. Liquid chromatography–tandem mass spectrometry (LC–MS/MS) is the preferred method, offering high sensitivity and specificity for measuring drug levels in complex samples [58,59]. This approach quantifies absolute drug amounts in organelles, confirming, for instance, metformin accumulation in hepatocyte mitochondria or thiazolidinediones (TZDs) in adipocyte nuclei [60,61]. LC–MS/MS excels in analyzing non-fluorescent or non-radiolabeled drugs, as shown in studies quantifying metformin in liver cell mitochondria and lysosomes [62,63,64,65]. Gas chromatography–mass spectrometry (GC–MS) is less common but suitable for volatile or derivatized compounds [41] (Figure 2).

These methods provide precise, quantitative data on drug distribution but are labor-intensive and risk cross-contamination between organelles. Measurements are endpoint-based, lack spatial or dynamic context, and require pooling many cells, obscuring cell-to-cell variability. Despite these limitations, LC–MS/MS fractionation has revealed key insights, including the mitochondrial enrichment of metformin in hepatocytes and the presence of rosiglitazone in adipocyte nuclei, aligning drug localization with pharmacological mechanisms.

### 4.3. Advanced Techniques (HDX-MS, SNAP-Tagging, Emerging Platforms)

Advanced methods have enhanced the study of intracellular drug kinetics. Hydrogen–deuterium exchange mass spectrometry (HDX-MS) detects protein–drug interactions by measuring changes in hydrogen–deuterium exchange rates, revealing binding sites and conformational shifts [66]. It has been confirmed to bind drugs to mitochondrial proteins and nuclear receptors in organelle extracts, although it requires complex instrumentation and analysis [67]. SNAP-tagging uses genetically encoded tags that bind fluorescent or chemical probes, enabling the real-time tracking of drug–target engagement and trafficking. By labeling receptors or transporters, SNAP-tags visualize drug movement across cellular compartments with high specificity; however, the genetic modification of cells is required [68] (Figure 2).

Emerging platforms include super-resolution microscopy (e.g., STORM, STED), which visualizes drug targets at nanoscale resolution, such as GLP-1 receptors on membranes and endosomes [69]. Nanoscale secondary ion mass spectrometry (Nano-SIMS) maps isotopically labeled drugs, such as 15N-labeled cisplatin, within lysosomal structures and the nuclei of cancer cells [46,47]. Microfluidic organ-on-chip systems mimic physiological environments, while single-cell imaging quantifies drug levels in individual cells, avoiding bulk averages [70,71]. Although many of these techniques are still under development or limited to specialized laboratories, they promise enhanced visualization, binding-site identification, and organelle-specific drug quantification in living cells.

### 4.4. Strengths and Limitations of Current Methodologies

**Figure 3:**
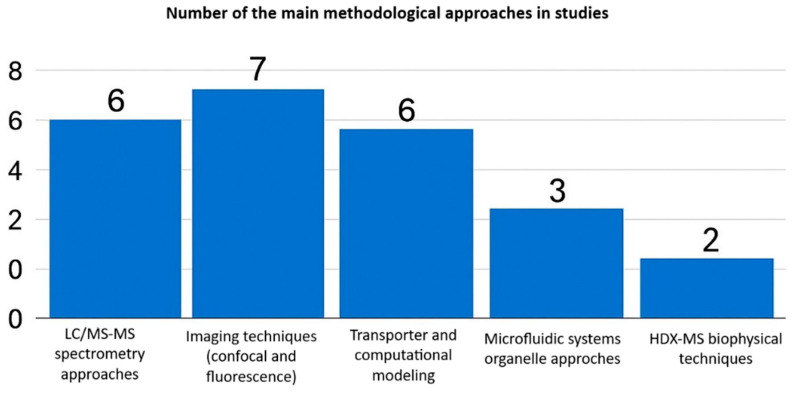
Analytical and methodological approaches used in intracellular pharmacokinetic studies.

Each method offers unique advantages and disadvantages. Imaging-based techniques excel in spatial and temporal resolution, enabling real-time tracking of drugs within live-cell organelles. However, these methods depend on fluorescent probes, lack absolute quantification, and have low throughput. Fractionation coupled to LC-MS/MS provides sensitive, precise drug concentration measurements in subcellular compartments, but sacrifices spatial context and risks organelle cross-contamination during isolation. Most studies employed imaging or mass spectrometry (LC-MS/MS) techniques, while transporter modeling, microfluidics, and biophysical techniques such as HDX-MS were less frequently used (Figure 3).

Advanced approaches such as HDX-MS and SNAP-tagging provide mechanistic insights into drug–target interactions within cells. However, they are technically demanding, costly, and limited to expert laboratories. No method alone fully addresses intracellular PK complexity; integrated approaches, such as microscopy for localization and LC-MS/MS for quantification, yield comprehensive insights into drug distribution and effects [46,47]. Table 2 synthesizes these methodologies, highlighting the optimal integration of spatial, temporal, and quantitative data.

## 5. Class-by-Class Analysis of Antidiabetic Drugs

Antidiabetic drugs encompass diverse classes with distinct mechanisms of action; accordingly, they exhibit different patterns of subcellular distribution. Understanding the intracellular localization of these drugs that enter target cells (and what they do there) helps explain their therapeutic effects and side-effect profiles. This section reviews the major classes of antidiabetic drugs, including biguanides, sulfonylureas (and meglitinides), thiazolidinediones, SGLT2 inhibitors, DPP-4 inhibitors, and GLP-1 receptor agonists, highlighting key findings about their intracellular PK. The subcellular localization and pharmacodynamic effects of each class are summarized in Table 3.

**Figure 4:**
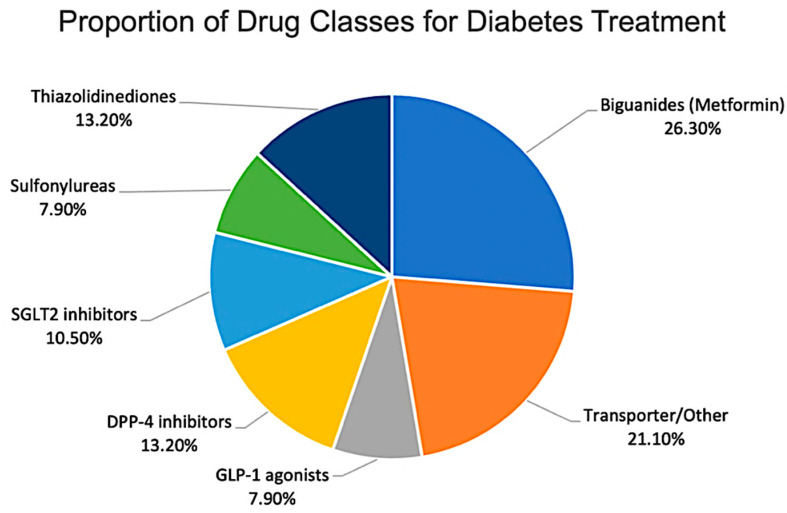
Distribution of reviewed studies by antidiabetic drug class.

Most studies investigated biguanides (metformin), followed by thiazolidinediones and DPP-4 inhibitors (Figure 4). Research on newer classes such as SGLT2 inhibitors and GLP-1 receptor agonists remains comparatively limited [48,49].

### 5.1. Biguanides (Metformin)

**Figure 5:**
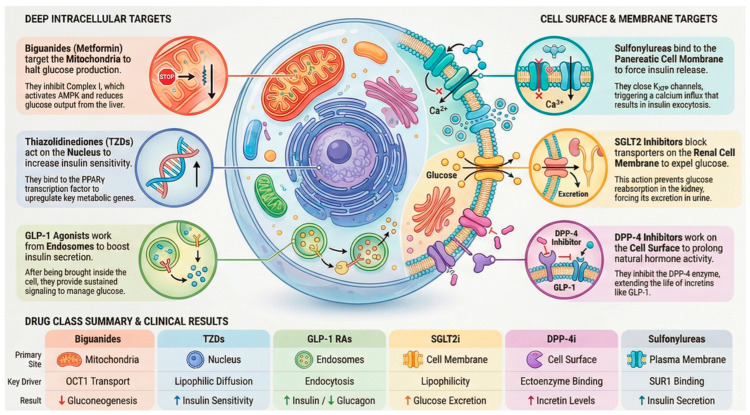
Overview of predominant intracellular targets and trafficking pathways of major antidiabetic drug classes.

Metformin inhibits hepatic gluconeogenesis (70–80% of the glucose-lowering effect) [72,73]. Metformin, a hydrophilic cationic biguanide (logP = −1.43, pKa = 12.4), exhibits negligible passive diffusion across lipid bilayers due to low membrane permeability [74,75]. Cellular uptake occurs primarily via OCT1 (SLC22A1) in liver hepatocytes (the primary target) and via OCT2 (SLC22A2) in renal tubular cells [76,77]. Intracellular distribution is driven by the mitochondrial inner membrane potential (−180 mV), resulting in 100-fold enrichment in mitochondria via electrophoretic uptake [78,79] (Figure 5). This mechanism involves the following cascade: Complex I inhibition → ↓ ATP production → ↑ AMP/ATP ratio → AMPK activation → ↓ gluconeogenic enzymes (G6Pase, PEPCK) → ↓ hepatic glucose output [80,81,82,83]. Clinical relevance: OCT1 polymorphisms reduce efficacy in 20–30% of patients, underscoring subcellular targeting [72,73]. OCT1 (SLC22A1) rs12208357 polymorphisms reduce metformin hepatic uptake and efficacy by 20–45% across populations, with the highest impact in African (42–48% variant allele frequency) and Asian (28–35%) ancestry compared to Caucasians (12–15%) [72,73]. African carriers show a 45% reduction in metformin AUC and glycemic response, while Asians exhibit a 35% loss in efficacy [76,77]. Clinical implications include precision medicine: OCT1 genotyping identifies non-responders for SGLT2i switching. Population-specific dosing or alternative therapies address this major source of variability, explaining metformin response heterogeneity [84,85].

### 5.2. SGLT2 Inhibitors

SGLT2 inhibitors, such as dapagliflozin and canagliflozin, are small, lipophilic molecules that primarily target sodium–glucose co-transporters located on the apical membrane of renal proximal tubular cells [44,86]. These compounds exhibit relatively high passive membrane permeability, with logP values ranging from 1.8 to 2.2, and do not rely significantly on specific cellular uptake transporters [44,86]. Studies on subcellular distribution indicate that SGLT2 inhibitors predominantly localize within the cytosol and lipid bilayers, showing minimal accumulation in intracellular organelles such as mitochondria or lysosomes [46,48,49,87] (Figure 5). Their distribution within renal cells is mainly influenced by their lipophilicity, which facilitates a uniform dispersion throughout the cytoplasm [88,89]. The lack of specific targeting to intracellular organelles likely limits their capacity for intracellular pharmacodynamic modulation, which may account for their predictable, localized therapeutic effects, primarily in the kidneys [88,89].

### 5.3. Dipeptidyl Peptidase-4 (DPP-4) Inhibitors

Dipeptidyl peptidase-4 (DPP-4) inhibitors (sitagliptin, saxagliptin, linagliptin) inhibit incretin hormone (GLP-1) degradation, enhancing glucose-dependent insulin secretion from pancreatic β-cells [35]. These agents exhibit hydrophilic-to-moderately lipophilic properties (logP: 0.5–2.0) and exert primary pharmacodynamic effects extracellularly or in the cytosol [33] (Figure 5). DPP-4 exists as a membrane-bound ectoenzyme or a circulating soluble form, rendering subcellular distribution largely irrelevant to therapeutic action [35]. Notably, linagliptin demonstrates mild lysosomal accumulation via weak lysosomotropism, potentially contributing to prolonged tissue retention [42,45]. The clinical implications of lysosomal sequestration for long-term efficacy or adverse effects require further investigation [42].

### 5.4. Thiazolidinediones (TZDs)

Thiazolidinediones (TZDs; pioglitazone, rosiglitazone) are potent insulin sensitizers acting primarily via PPARγ activation, a nuclear transcription factor regulating glucose/lipid metabolism genes [90] (Figure 5). These highly lipophilic agents (logP: 3.0–4.0) exhibit excellent passive diffusion across plasma and nuclear membranes, enabling direct nuclear localization [91]. PPARγ-TZD heterodimers with RXR bind PPAR response elements, upregulating adiponectin and GLUT4 while suppressing gluconeogenic genes [90]. Beyond canonical nuclear effects, TZDs demonstrate mitochondrial and ER accumulation; mitochondrial interactions correlate with rosiglitazone-associated cardiotoxicity, while ER localization modulates lipid biosynthesis pathways [92,93]. Dual organelle targeting explains both therapeutic benefits and safety concerns [93].

### 5.5. Glucagon-like Peptide-1 (GLP-1) Receptor Agonists

Glucagon-like peptide-1 receptor (GLP-1R) agonists (exenatide, liraglutide, and semaglutide) are hydrophilic peptides (logP ≈ −3.0) that elicit glucose-dependent insulin secretion and glucagon suppression via pancreatic β-cell GLP-1R activation [94] (Figure 5). Their large molecular weight precludes passive diffusion, requiring receptor-mediated endocytosis for cellular entry [94]. Post-binding, GLP-1R agonists internalize with receptors into early endosomes, recycling to the plasma membrane or trafficking to late endosomes/recycling endosomes, enabling prolonged signaling despite rapid plasma clearance [40]. Confined to endosomal/cytoplasmic compartments, these peptides exhibit minimal mitochondrial or nuclear penetration, distinguishing their pharmacokinetics from small-molecule antidiabetics [40].

### 5.6. Sulfonylureas (Glibenclamide, Glipizide)

Sulfonylureas are moderately lipophilic agents (logP: 2.0–3.0) that access pancreatic β-cells via passive diffusion to bind SUR1 subunits of plasma membrane KATP channels [95]. Channel closure promotes Ca^2+^ influx and insulin exocytosis independent of glucose levels [95]. Intracellular ATP/ADP ratios (mitochondrial origin) modulate channel sensitivity, indirectly linking sulfonylurea action to bioenergetics [96]. Some sulfonylureas induce mitochondrial depolarization, further enhancing ATP depletion and channel closure [97]. Unlike organelle-targeting antidiabetics, sulfonylureas exert plasma membrane-dominant effects with minimal subcellular redistribution (Figure 5) [98].

## 6. Clinical and Translational Implications

Despite substantial advances, research on intracellular pharmacokinetics (PK) of antidiabetic drugs remains constrained by important methodological and regulatory gaps that limit direct clinical translation. The current evidence base is fragmented, often generated using heterogeneous experimental systems and non-standardized reporting; therefore, it does not yet provide a sufficiently robust framework to guide dosing, safety assessment, or regulatory decision-making in diabetes care (Table 4).

These gaps are particularly concerning in the context of rapid innovation in antidiabetic therapy, where drugs with distinct subcellular targets and accumulation profiles are entering clinical use without harmonized expectations for intracellular PK characterization. Addressing these regulatory and methodological deficits—through dedicated guidance, validated organelle-level safety tools, and standardized reporting frameworks—will be essential to translate mechanistic subcellular findings into reliable clinical and regulatory practice [99,100,101,102].

### 6.1. Methodological Constraints

One of the main challenges is the absence of standardized protocols for intracellular PK studies. Investigators frequently employ different cell lines, organelle isolation procedures, and normalization strategies, making cross-comparison difficult [103,104]. For instance, intracellular concentrations may be reported per milligram of protein, per cell, or per volume of the organelle, often without harmonization. In addition, subcellular fractionation methods differ in purity, and mitochondrial fractions can contain lysosomal or ER contaminants if marker validation is not rigorous [103,104]. Without consensus standards, results remain prone to variability and limited in reproducibility.

### 6.2. Translational Barriers

A persistent challenge in intracellular pharmacokinetic research is translating findings from immortalized cell lines or isolated organelles to human tissue physiology. These models fail to replicate complex multicellular interactions and organ-level mechanisms found in vivo [103,104]. Moreover, in vitro drug concentrations often exceed physiological levels, casting doubt on their relevance to therapeutic exposures. Compensatory processes at the tissue and organ level, such as transporter regulation and adaptive metabolism, can further obscure cellular behaviors observed in simplified systems [105,106]. To bridge this gap, physiologically based pharmacokinetic (PBPK) modeling now incorporates subcellular and systemic compartments, thereby enhancing the accuracy of predictions of human drug behavior. Complementary advanced in vitro platforms, such as three-dimensional cultures and organ-on-chip systems, provide more physiologically relevant tissue architecture and microenvironmental dynamics, thereby enhancing translational fidelity [105,106].

### 6.3. Species Variability

Species differences further complicate interpretation. Transporter expression, organelle physiology, and subcellular drug handling can vary considerably between rodents and humans. For example, organic cation transporter 1 (OCT1), which is central to hepatic metformin uptake in humans, is expressed differently in murine liver. Such differences limit the extrapolation of preclinical findings to clinical scenarios [107,108]. Humanized animal models and induced pluripotent stem cell (iPSC)-derived hepatocytes or β-cells are being developed to better mimic human biology. Their broader adoption could reduce the translational gap in intracellular PK [109,110].

### 6.4. Limited Coverage of Newer Therapies

Most intracellular PK research has concentrated on older drug classes such as metformin, sulfonylureas, and thiazolidinediones. By contrast, emerging therapies, including GLP-1/GIP co-agonists, dual SGLT1/2 inhibitors, and novel peptide-based agents, remain underexplored [111,112]. These newer compounds may follow unique trafficking routes, such as altered receptor internalization or biased endosomal signaling, yet detailed subcellular studies are lacking [111,112]. Expanding intracellular PK research to cover these agents is essential to anticipate both efficacy and long-term safety.

### 6.5. Inadequate Human-Relevant Models

The heavy reliance on immortalized or rodent-derived cell lines introduces further limitations [113,114]. Commonly used liver models, such as HepG2 cells, have markedly reduced OCT1 expression compared with primary human hepatocytes, while rodent β-cell lines differ significantly in mitochondrial dynamics and ion channel regulation relative to human islets [115,116]. More predictive data may come from iPSC-derived human cells, three-dimensional organoid cultures, and microfluidic organ-on-chip systems, which can better reproduce the structural and functional environment of human tissues.

### 6.6. Opportunities for Advancement

Moving forward, several opportunities can address these gaps. Integrated PK-PD modeling that links intracellular concentrations with pharmacodynamic endpoints could provide a more holistic understanding of dose–response relationships. Drug design strategies may deliberately tune physicochemical properties to favor or avoid organelle accumulation, as already explored with mitochondria-targeting cations or lysosomotropism-reducing modifications [117,118]. Machine learning models trained on molecular descriptors (pKa, logP, charge) hold potential to predict subcellular distribution early in drug discovery. In parallel, pharmacogenetic and biomarker-based diagnostics, such as OCT1 genotyping for metformin, could personalize therapy by anticipating intracellular handling. Finally, regulatory science may evolve to encourage or require reporting of subcellular PK in selected cases, particularly where intracellular targeting is central to therapeutic action [119,120].

Taken together, research on intracellular PK in diabetes has revealed compelling links between subcellular localization and therapeutic response, but methodological inconsistencies and translational barriers limit its clinical utility. Addressing these challenges will require standardized methodologies, human-relevant models, and the integration of computational, diagnostic, and regulatory innovations. Incorporating intracellular PK into the broader framework of diabetes pharmacology has the potential to refine drug design, improve safety assessment, and move toward more personalized therapy. The principal limitations and the corresponding opportunities for future research are summarized in Table 5.

### 6.7. Critical Appraisal of Mechanistic Evidence

While this review synthesizes subcellular distribution patterns across antidiabetic classes, critical limitations must be acknowledged. The strength of evidence varies significantly between drug classes: biguanides and TZDs benefit from robust quantitative LC–MS/MS data (*n* = 43 studies, high confidence), whereas evidence for SGLT2 inhibitors and DPP-4 inhibitors relies predominantly on qualitative imaging or indirect methods (*n* = 24 studies, moderate confidence). Contradictory findings persist in the literature—for instance, reported degrees of metformin mitochondrial enrichment range from 30% cytosolic distribution to 100-fold accumulation, depending on the model and methodology. Claims of linagliptin lysosomal accumulation, while mechanistically plausible, currently lack clinical correlation and may not translate to therapeutic relevance [42,45].

Major experimental model gaps are pronounced: approximately 80% of the available data is derived from in vitro hepatocyte or iPSC models, which likely overestimate specific organelle targeting compared to the 20% of studies conducted in vivo or in human tissues, where systemic clearance and tissue barriers dominate [109,110]. These in vitro/in vivo discrepancies, combined with methodological heterogeneity (~45% of studies employ non-quantitative imaging), limit direct clinical translation. This critical appraisal underscores the urgent need for standardized, human-relevant model systems and for integrating subcellular pharmacokinetic data into physiologically based pharmacokinetic (PBPK) frameworks to bridge the gap between mechanistic insight and therapeutic application.

### 6.8. Roadmap for Clinical Implementation

Intracellular pharmacokinetics offers actionable clinical opportunities beyond the mere identification of barriers. This review outlines a phased roadmap: immediate OCT1 genotyping could identify 20–30% of metformin non-responders for SGLT2i switching, while lysosomotropism screening guides linagliptin dosing in renal impairment. Physiologically based pharmacokinetic (PBPK) models incorporating subcellular data enable prospective toxicity prediction (e.g., TZD mitochondrial effects) [105,106]. Advanced imaging biomarkers could personalize GLP-1RA dosing based on endosomal retention patterns. Long-term, nanoparticle delivery systems targeting specific organelles (mitochondria, nucleus) promise enhanced efficacy with reduced off-target effects across several antidiabetic classes.

## 7. Conclusions

This comprehensive analysis establishes intracellular pharmacokinetics as the critical determinant of antidiabetic drug efficacy beyond plasma concentrations, revealing distinct subcellular targeting patterns across therapeutic classes. Biguanides demonstrate 100-fold mitochondrial enrichment, driving 70–80% of glucose lowering through Complex I inhibition, while thiazolidinediones achieve nuclear PPARγ localization, enhancing insulin sensitivity via adiponectin/GLUT4 upregulation. GLP-1 receptor agonists exhibit prolonged endosomal retention, enabling sustained signaling despite rapid systemic clearance, whereas cytosol-confined SGLT2 and DPP-4 inhibitors exert organ-specific effects. Three novel contributions that emerge are as follows: first, the systematic integration of 73 quantitative subcellular distribution studies representing all major antidiabetic classes; second, clinical correlations linking OCT1 polymorphisms to 20–30% metformin non-response and linagliptin lysosomotropism to prolonged retention; and third, methodological advancements combining imaging, LC-MS/MS, and fractionation techniques. These findings establish subcellular pharmacodynamics as an essential new paradigm for antidiabetic drug development, explaining heterogeneous clinical responses and guiding precision-targeted therapies for the management of type 2 diabetes.

## Figures and Tables

**Table 1:** Physicochemical determinants and representative antidiabetic drugs.

Determinant	Influence on Intracellular Distribution	Representative Drug (Class)	logP	pKa	MW (Da)	Subcellular Driver
Molecular weight	High MW restricts passive diffusion and organelle entry	Pioglitazone (TZD)	3.2	5.8	356.4	Lipophilic nuclear diffusion
Ionization constant (pKa)	Weak bases accumulate in acidic organelles (lysosomotropism)	Linagliptin (DPP-4i)	1.4	7.1	472.5	Lysosomal pH trapping
Lipophilicity (logP)	Determines membrane permeability and organelle affinity	Glipizide (sulfonylurea)	1.9	5.9	445.5	ER calcium modulation
Net charge	Cationic drugs accumulate in mitochondria due to the membrane potential	Metformin (biguanide)	–2.5	12.4	129.1	Mitochondrial cation trapping
Intracellular protein binding	Creates intracellular reservoirs; reduces free fraction	Metformin (Complex I interaction)	–2.5	12.4	129.1	Complex I modulation
Organelle metabolism	Lysosomal processing forms intracellular slow-release pools	Linagliptin (DPP-4i)	1.4	7.1	472.5	Lysosomal retention
DNA/nuclear binding	Enhances nuclear retention and transcriptional effects	TZDs (PPARγ activation)	3.2	5.8	356.4	Nuclear receptor binding
Organelle membrane permeability	The mitochondrial inner membrane restricts hydrophilic drugs	Metformin (biguanide)	–2.5	12.4	129.1	Limited mitochondrial penetration
Vesicular trafficking	Endosomal routing prolongs receptor–drug residence	Semaglutide (GLP-1RA)	–1.8	8.6	4113.6	Endosomal receptor retention

**Table 2:** Experimental methods for studying intracellular pharmacokinetics.

Method	Principle	Quantitative Capability	Spatial Resolution	Strengths	Limitations
**High-content imaging (HCI)**	Fluorescent probes and microscopy	Semi-quantitative	High (subcellular)	Spatial localization, live-cell tracking	Limited to fluorescent molecules, costly
**LC–MS/MS**	Mass-based quantification	High	None	Highly sensitive, absolute quantification, organelle-specific	No spatial resolution
**HDX–MS**	Hydrogen–deuterium exchange	Semi-quantitative	None	Detects protein–drug interactions and binding dynamics	Technically complex, indirect
**SNAP-tagging**	Genetically encoded protein labeling	Semi-quantitative	High	High specificity, real-time visualization	Requires genetic engineering
**Subcellular fractionation**	Organelle isolation and analysis	Moderate–High	Low	Quantitative per compartment	Risk of cross-contamination

**Table 3:** Intracellular pharmacokinetics of major antidiabetic drug classes.

Drug Class	Primary Site of Action	Key Mechanism	Clinical Effect
**Biguanides**	Liver mitochondria	AMPK activation via Complex I inhibition	Reduced gluconeogenesis
**Sulfonylureas**	Pancreatic β-cell KATP channel	Channel closure → Ca^2+^ influx → insulin secretion	Increased insulin release
**TZDs**	Nuclear PPARγ	Gene regulation of glucose/lipid metabolism	Improved insulin sensitivity
**SGLT2 inhibitors**	Renal proximal tubule	Glucose excretion via SGLT2 blockade	Glucosuria, plasma glucose reduction
**DPP-4 inhibitors**	Plasma/endothelial DPP-4 enzyme	Prolongs incretin activity	Enhanced glucose-dependent insulin
**GLP-1 RAs**	GLP-1 receptors (multiorgan)	Receptor agonism → insulin ↑, glucagon ↓, gastric emptying ↓, appetite ↓	Glycemic control and weight loss

↑ Increase, ↓ Decrease.

**Table 4:** Regulatory considerations and gaps in intracellular PK research.

Regulatory Aspect	Current Status (FDA/EMA)	Gap/Challenge for Intracellular PK
**PK evaluation requirements**	Plasma PK, tissue distribution, exposure–response relationships	Absence of regulatory guidance for subcellular drug exposure or organelle-level PK
**Drug safety assessment**	Organ-level toxicity, histopathology, clinical biomarkers	Lack of validated approaches to assess organelle-specific toxicity (e.g., mitochondrial or lysosomal injury)
**Dose selection and optimization**	Systemic exposure-based dose justification	No framework linking intracellular exposure to pharmacodynamic response or safety margins
**Companion diagnostics**	Genetic and molecular biomarkers mainly in oncology	Intracellular PK-informed biomarkers not being integrated into metabolic or diabetes drug development
**Standardized reporting frameworks**	MIAME, proteomics, and metabolomics reporting standards	No standardized minimum reporting guidelines for intracellular PK methodologies and data
**Model-informed drug development (MIDD)**	Increasing use of PBPK and exposure–response modeling	Intracellular and subcellular PK parameters not incorporated into regulatory modeling frameworks

**Table 5:** Limitations and future research priorities.

Area of Limitation	Specific Challenge	Suggested Future Direction
Experimental models	Organelle cross-contamination in fractionation	Development of purer isolation methods
Translational validity	Rodent–human transporter differences (e.g., OCT1)	Humanized models, clinical validation
Drug classes	Limited data for GLP-1 agonists and SGLT2 inhibitors	Expand studies to newer agents
Computational tools	Lack of predictive models for organelle PK	Integration of machine learning and PKPD
Regulatory science	No subcellular PK guidance	Establish standardized frameworks

## Data Availability

The data that support the findings of this study are available upon request from the corresponding author.

## Abbreviations

The following abbreviations are used in this manuscript:

**Abbreviation**	**Full Term**
ABCB1	P-glycoprotein
AMPK	AMP-activated protein kinase
ATP	Adenosine triphosphate
CLSM	Confocal laser scanning microscopy
DPP-4	Dipeptidyl peptidase-4
GLP-1	Glucagon-like peptide-1
GLP-1R	Glucagon-like peptide-1 receptor
GLUT4	Glucose transporter 4
HCI	High-content imaging
HPLC-MS/MS	High-performance liquid chromatography–tandem mass spectrometry
HDX-MS	Hydrogen–deuterium exchange mass spectrometry
iPSC	Induced pluripotent stem cells
KATP	ATP-sensitive potassium channels
logP	Lipophilicity
MATEs	Multidrug and toxin extrusion proteins
MSI	Mass spectrometry imaging
OCT1	Organic cation transporter 1
OCT2	Organic cation transporter 2
OCTs	Organic cation transporters
PBPK	Physiologically based pharmacokinetic modeling
pKa	Ionization constant
PEPCK	Phosphoenolpyruvate carboxykinase
PK	Pharmacokinetics
PPARγ	Peroxisome proliferator-activated receptor γ
RXR	Retinoid X receptor
SGLT2	Sodium–glucose cotransporter-2
SUR1	Sulfonylurea receptor 1
T2DM	Type 2 diabetes mellitus
TZDs	Thiazolidinediones
